# Neprilysin inhibitors and risk of Alzheimer's disease: A future perspective

**DOI:** 10.1111/jcmm.17993

**Published:** 2023-10-17

**Authors:** Naif H. Ali, Hayder M. Al‐Kuraishy, Ali I. Al‐Gareeb, Saud A. Alnaaim, Athanasios Alexiou, Marios Papadakis, Asmaa A. Khalifa, Hebatallah M. Saad, Gaber El‐Saber Batiha

**Affiliations:** ^1^ Department of Internal Medicine, Medical College Najran University Najran Saudi Arabia; ^2^ Department of Clinical Pharmacology and Medicine, College of Medicine Mustansiriyah University Baghdad Iraq; ^3^ Clinical Neurosciences Department, College of Medicine King Faisal University Hofuf Saudi Arabia; ^4^ Department of Science and Engineering Novel Global Community Educational Foundation Hebersham New South Wales Australia; ^5^ AFNP Med Wien Austria; ^6^ Department of Surgery II University Hospital Witten‐Herdecke, University of Witten‐Herdecke Wuppertal Germany; ^7^ Department of Pharmacology and Therapeutics, Faculty of Pharmacy Pharos University in Alexandria Alexandria Egypt; ^8^ Department of Pathology, Faculty of Veterinary Medicine Matrouh University Matrouh Egypt; ^9^ Department of Pharmacology and Therapeutics, Faculty of Veterinary Medicine Damanhour University Damanhour AlBeheira Egypt

**Keywords:** Alzheimer's disease, neprilysin, neprilysin inhibitors

## Abstract

Alzheimer's disease (AD) is a heterogeneous neurodegenerative disease with multifaceted neuropathological disorders. AD is characterized by intracellular accumulation of phosphorylated tau proteins and extracellular deposition of amyloid beta (Aβ). Various protease enzymes, including neprilysin (NEP), are concerned with the degradation and clearance of Aβ. Indeed, a defective neuronal clearance pathway due to the dysfunction of degradation enzymes might be a possible mechanism for the accumulation of Aβ and subsequent progression of AD neuropathology. NEP is one of the most imperative metalloproteinase enzymes involved in the clearance of Aβ. This review aimed to highlight the possible role of NEP inhibitors in AD. The combination of sacubitril and valsartan which is called angiotensin receptor blocker and NEP inhibitor (ARNI) may produce beneficial and deleterious effects on AD neuropathology. NEP inhibitors might increase the risk of AD by the inhibition of Aβ clearance, and increase brain bradykinin (BK) and natriuretic peptides (NPs), which augment the pathogenesis of AD. These verdicts come from animal model studies, though they may not be applied to humans. However, clinical studies revealed promising safety findings regarding the use of ARNI. Moreover, NEP inhibition increases various neuroprotective peptides involved in inflammation, glucose homeostasis and nerve conduction. Also, NEP inhibitors may inhibit dipeptidyl peptidase 4 (DPP4) expression, ameliorating insulin and glucagon‐like peptide 1 (GLP‐1) levels. These findings proposed that NEP inhibitors may have a protective effect against AD development by increasing GLP‐1, neuropeptide Y (NPY) and substance P, and deleterious effects by increasing brain BK. Preclinical and clinical studies are recommended in this regard.

## 1 INTRODUCTION

Alzheimer's disease (AD) is a heterogeneous neurodegenerative disease characterized by memory loss and cognitive dysfunction.^1^ AD is the most common type of dementia, accounting for about 70% of all dementia types.^1^ Most AD cases, along with sporadic forms of the disease that develop after the age of 65 years are called late‐onset AD. In contrast, 5% of AD cases are genetically typed due to a mutation of the amyloid precursor protein (APP) gene developed in early‐onset AD.^2^ AD is the seventh leading cause of death in the United States, affecting 50 million people globally.^3^ Approximately 6% of the general population is affected, whereas more than 65% of affected cases are women; nonetheless, 10% of early‐onset dementia affecting people aged 30–60 is attributed to AD.^3^

AD is characterized by intracellular accumulation of phosphorylated tau proteins as neurofibrillary tangles (NFTs) and extracellular deposition of amyloid beta (Aβ) as neuritic plaques.^4^ In AD, extracellular deposition of Aβ, mainly Aβ1‐42 in the neocortex and hippocampus leads to dementia and cognitive decline.^4^, ^5^ Moreover, different mechanisms are also proposed in AD pathogenesis, including inflammation, oxidative stress, cholinergic dysfunction and impairment of the melatonin pathway.^4^, ^5^

It has been suggested that Aβ plaques play a critical role in the sequestration of the soluble form of Aβ to reduce its neurotoxic effect. Afterwards, plaque sequestration capacity is reduced with AD progression, and soluble Aβ can diffuse extracellularly, causing extensive synaptic dysfunction and neuronal injury.^6^ Aβ oligomers also trigger intracellular and extracellular neurotoxicity through interaction with cell membrane ion channels and receptors. These pathological changes promote a profound imbalance between inhibitory and excitatory neurotransmitters with the development of hyper‐excitability.^7^ In turn, hyper‐excitability contributes to the deposition of Aβ and the progression of neurodegeneration.^8^, ^9^, ^10^, ^11^, ^12^ An imaging study and computational neuronal modelling involving AD patients demonstrated significant subpopulation alteration in the excitatory/inhibitory axis concerning the severity of Aβ deposition compared to the controls.^13^ Consequently, synaptic dysfunction and developed excitatory/inhibitory imbalance promote Aβ‐induced neuronal injury and AD progression.^14^ These changes influence the lysosomal and endosomal clearance pathways with the development of synaptic dysfunction and the formation of senile amyloid plaques.^15^

**FIGURE 1:**
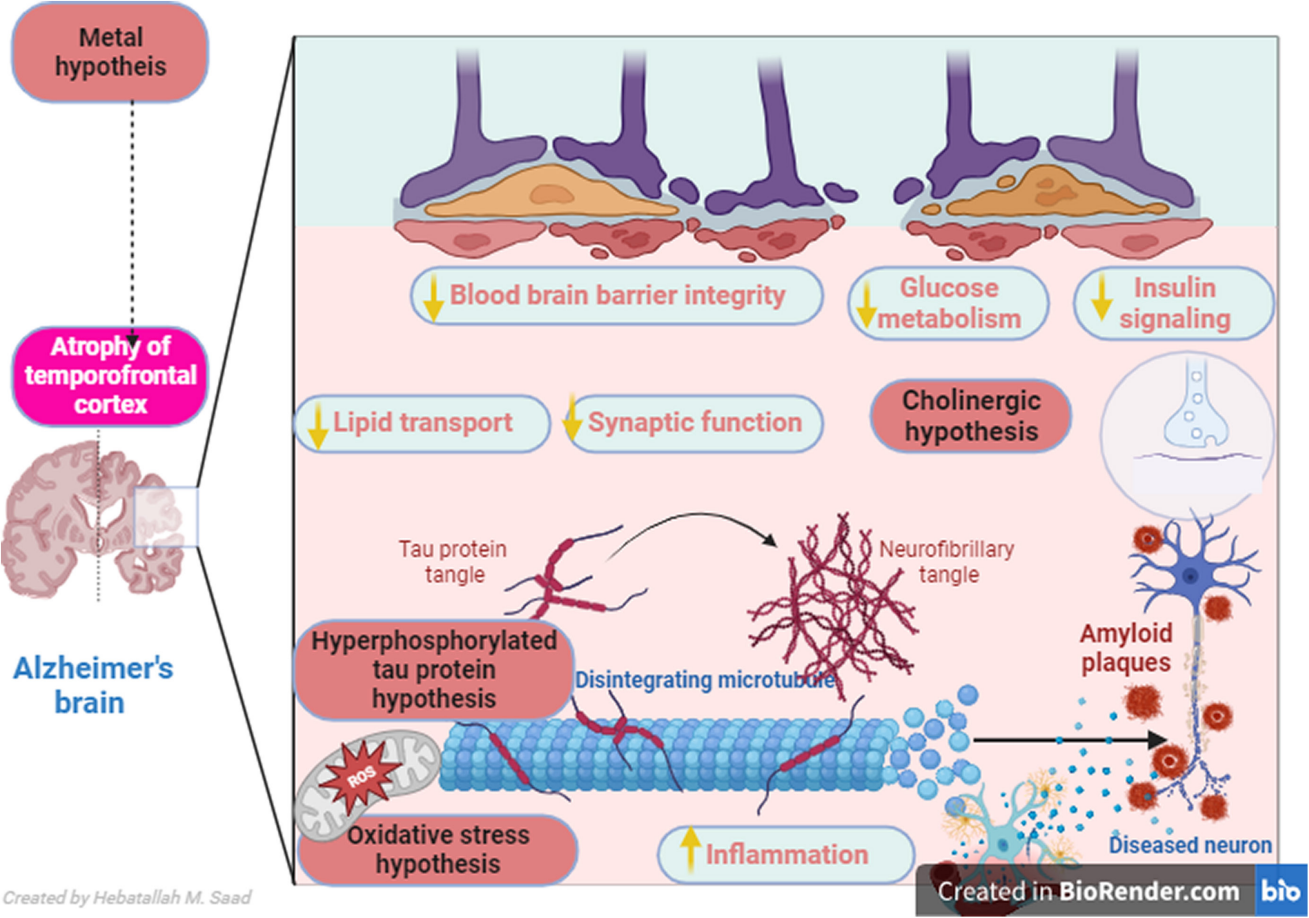
Mechanistic pathways underlying Alzheimer's disease (AD): Impairment of blood–brain barrier (BBB), brain glucose metabolism and dysregulation of brain insulin signalling lead to deterioration of brain lipid metabolism and synaptic dysfunction mainly in cholinergic neurons. These pathological alterations induce tau protein hyperphosphorylation with the accumulation of amyloid plaques, oxidative stress and neuroinflammation. As well, metal hypotheses suggest that the accumulation of metals triggers atrophy of the temporofrontal cortex and the development of AD.

The accumulation of Aβ is a central point in AD pathogenesis, causing neuronal injury and synaptic loss.^2^ Aβ42 has a higher propensity to aggregate and form Aβ plaques.^2^ Accumulation of Aβ in the brain does not depend on the synthesis from APP only but also due to defects in the proteolytic degradation and clearance of Aβ itself, which occur mainly in late‐onset AD.^16^ Indeed, a defective neuronal clearance pathway due to the dysfunction of degradation enzymes might be a possible mechanism for accumulating Aβ and NFTs with subsequent propagation of AD neuropathology.^17^ Various proteases enzymes like matrix metalloproteinase 9 (MMP‐9), endothelin converting enzyme, neprilysin (NEP), insulin‐degrading enzyme and plasmin are concerned in the degradation and clearance of Aβ and NFTs.^2^, ^18^ These findings suggest that Aβ accumulation and release of soluble Aβ with defective clearance capacity are involved in AD neuropathology (Figure 1). Therefore, objective of the present review was to clarify the possible role of NEP and NEP inhibitors on the cognitive function and AD risk.

## 2 NEP AND Aβ CLEARANCE IN AD

**FIGURE 2:**
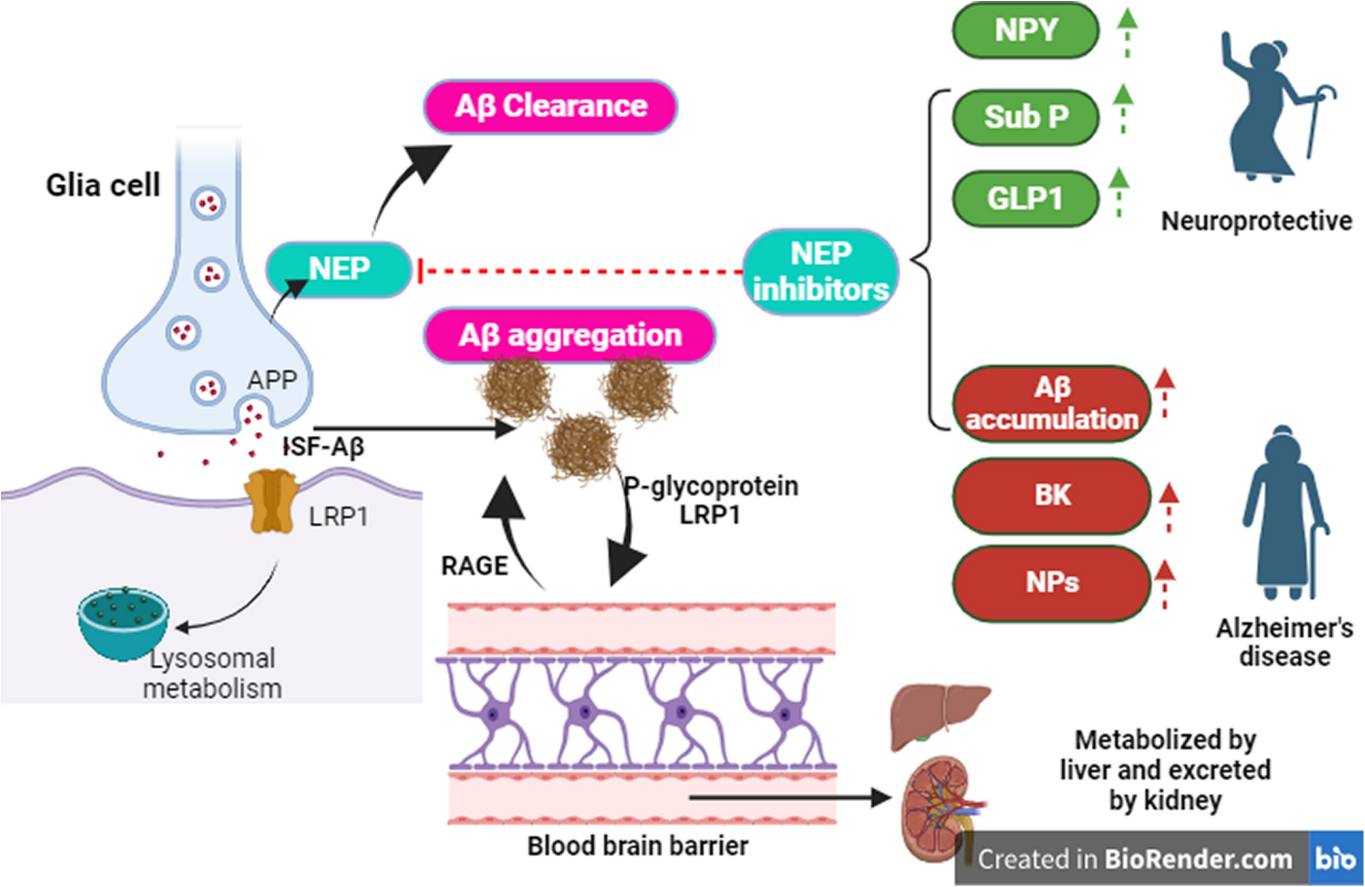
Transport and metabolism of amyloid beta (Aβ): Presynaptic of glial cells promote the synthesis of amyloid beta (Aβ) from amyloid precursor protein (APP). Aβ is released into the synaptic cleft, and the Aβ in the interstitial fluid (ISF) is transported to postsynaptic neurons via lipoprotein receptor‐related protein 1 (LRP1) that is metabolized by lysosomes. However, some ISF‐Aβ can cross the blood–brain barrier (BBB) and be released into systemic circulation through P‐glycoprotein and lipoprotein receptor‐related protein 1 (LRP1) that is metabolized by the liver and excreted by the kidney. Re‐entry of Aβ from blood to the brain is mediated by a receptor of advanced glycation end‐product (RAGE). Also, NEP can metabolize Aβ. NEP inhibitors may have neuroprotective effects by increasing neuropeptide Y (NPY), substance P (sub P), and glucagon‐like peptide 1 (GLP‐1) or induce Alzheimer's disease (AD) neuropathology by increasing Aβ accumulation, bradykinin (BK) and natriuretic peptides (NPs).

Generated Aβ in the brain can transport across the blood–brain barrier (BBB) via P‐glycoprotein and lipoprotein receptor‐related protein 1 (LRP1) into blood circulation. Re‐entry of Aβ from blood to the brain is mediated by a receptor of advanced glycation end‐product (RAGE).^16^ Circulating Aβ is metabolized by the liver and excreted by the kidney.^16^, ^19^ APP is degraded by proteolytic enzymes in presynaptic neurons and then released to the synaptic cleft. The insoluble form of Aβ increases Aβ aggregates; however, sometimes they are up took by glial cells, bound to lipoprotein receptor‐related protein (LRP1) and metabolized by the lysosome in the postsynaptic neurons.^20^ These annotations proposed a balance regulation of brain Aβ through systemic circulation and metabolism (Figure 2).

In the brain, phagocytosis and lysosomal degradation of Aβ by microglial cells are concerned with the clearance of Aβ deposit.^20^ Therefore, microglial dysfunction is implicated in AD pathogenesis.^20^ Moreover, metalloproteinase enzymes can remove intracellular and extracellular Aβ by different mechanisms. Intracellular metalloproteinase enzymes in the secretory pathway degrade intracellular Aβ40 before its secretion, whereas extracellular metalloproteinase enzymes degrade extracellular Aβ42.^21^, ^22^ Thus, Aβ is not cleared by a single metalloproteinase enzyme; in this state, cooperation between intracellular and extracellular metalloproteinase enzymes is required to clear Aβ effectively.^23^ Remarkably, metalloproteinase enzymes have higher abilities to degrade monomeric Aβ but with a limited capacity to cleave fibrillar and oligomeric Aβ.^23^

NEP is one of the most imperative metalloproteinase enzymes involved in the clearance of Aβ.^23^ Iwata et al.^24^ observed that localization of NEP in presynaptic neurons facilitates the clearance of Aβ. A local increase of Aβ is associated with synaptic dysfunction in AD. Increasing expression of the NEP gene by recombinant adeno‐associated viral vector attenuates the increment of Aβ level in the hippocampus of NEP‐deficient mice.^24^ Different preclinical studies confirmed the ability of NEP to cleave Aβ and have a beneficial effect against AD neuropathology. NEP has been reported to cleave 27% of monomeric Aβ42 and 73% of monomeric Aβ40.^25^, ^26^ However, mutated Aβ are highly resistant to the proteolytic activity of NEP.^26^ In addition, intra‐neuronal Aβ42 deposit is highly cleaved by NEP in mice.^27^ Using transgenic *Drosophila* expressing human NEP and Aβ42, demonstrated that NEP efficiently suppressed the formation of intra‐neuronal Aβ42 deposits and Aβ42‐induced neuron loss.^27^ However, overexpression of neuronal NEP reduced cyclic adenosine monophosphate (cAMP)‐responsive element‐binding protein‐mediated transcription, caused age‐dependent axon degeneration and shortened the life span of the flies. Downregulation of NEP activity in aging brains may be an evolutionarily conserved phenomenon, which could predispose humans to developing late‐onset AD.^27^ A deficiency in NEP accelerates the formation of extracellular Aβ deposits, Aβ angiopathy, synaptic dysfunctions and memory deficits in transgenic mice.^28^ Activation of NEP could be a potential disease‐modifying therapy for AD. Delivery of NEP to brains of APP transgenic mice reduces extracellular Aβ deposits, synaptic dysfunction and premature death.^29^ Thus, the downregulation of NEP is associated with the progression of AD neuropathology. Poirier et al.^30^ revealed that higher expression of NEP was associated with a lower incidence of spatial memory deficit and development of AD in mice. It has been shown that upregulation of neuronal NEP leads to increased degradation of Aβ, and reduced the formation of Aβ‐plaques and the associated cytopathology, but whether overexpression of NEP can improve cognition is unknown.^30^ Neuronal overexpression of NEP improved the Morris water maze memory performance in mice with memory deficits resulting from overexpression of APP.^30^ This improvement was associated with decreased brain levels of Aβ and with unchanged endoproteolytic processing of APP.^30^ Decreased NEP levels in aged rodent brains contribute to the onset and progression of late‐onset AD.^31^ These results provide the evidence that lowering of brain Aβ levels by increasing its degradation can improve cognitive functions in vivo, and suggest that augmenting the activity of NEP in brain may be effectual in preventing cognitive decline in AD. Experiment studies revealed that NEP protein levels were reduced in some brain regions, including the cerebral cortex and hippocampus.^27^, ^32^ Downregulation of NEP activity contributes to cerebral amyloid angiopathy (CAA) progress. Homogenates of the frontal cortex of AD and controls illustrated that NEP activity was significantly decreased in the tissues of AD brains.^33^ Loss of vessels in the cerebral cortex correlated with a reduction of NEP activity.^33^ Reduction of NEP activity is not secondary to CAA but a primary event for the development and progression of CAA and AD neuropathology.^33^

Furthermore, clinical studies established the protective role of NEP in AD by increasing clearance of Aβ.^31^ In patients with late‐onset AD, NEP mRNA and protein levels are selectively reduced in brain regions extremely vulnerable to AD pathology,^31^ and NEP levels and activity decrease in human and rodent brains with aging, suggesting that reduction in NEP levels may contribute to the development of late‐onset AD.^32^ Thus, overexpression of NEP can decrease the risk of AD by reducing intra‐neuronal Aβ accumulation in patients following traumatic brain injury.^34^ In this state, polymorphism of NEP is associated with the risk of AD development.^34^ An increase in NEP immunoreactivity was observed in association with Aβ accumulation for up to 3 years following trauma.^34^ Other studies found no association between polymorphism of NEP and AD development.^35^ In addition, the activity of NEP could be age‐dependent related activity; it appears to be decreased with age, as confirmed by human study.^27^, ^32^ No difference in NEP mRNA and protein levels was perceived between AD patients and matched controls. However, NEP protein levels were reduced in both AD and controls frontal and temporal cortexes.^32^ Remarkably, a significant positive correlation between Aβ accumulations with age is found in aged control compared to AD patients due to age‐dependent clearance pathway dysfunction.^32^ No difference in NEP protein level was found between AD subjects and age‐matched controls. A significant positive correlation between insoluble Aβ_40_ and Aβ_42_ with age was found in the cortex of normal brain whereas in AD brain the correlation between age and Aβ was weaker.^32^ Thus, an inverse correlation between NEP and insoluble Aβ levels in both groups suggests that NEP is involved in the clearance of Aβ. The observed age‐dependent decline in NEP may be related to the increased Aβ levels during normal aging. The similar rate of decline in NEP with age may not be the major cause of the high levels of Aβ associated with AD but is likely to be a trigger of AD pathology.^32^ Accordingly, an age‐dependent decline in NEP activity triggers Aβ accumulations with activation of AD neuropathology. It has been shown that NEP protein level and its activity are reduced in the early phase of AD neuropathology and negatively correlated with Aβ load.^33^ Besides, NEP activity is also reduced in the CSF of the patients with cognitive impairments.^36^ CSF NEP level is reduced and inversely correlated with CSF Aβ_42_ level in AD patients.^36^ CSF‐NEP is well‐matched with the notion that local degradation, among other mechanisms of Aβ clearance, plays a role in the development of AD pathology. In addition, CSF‐NEP is associated with the extent and the rate of neurodegeneration.^36^ Therefore, a low CSF NEP level could reflect the severity of AD neurodegeneration.

In addition, different studies have revealed an inverse relationship between Aβ accumulations and CSF NEP levels in the cerebral cortex.^23^, ^32^ These findings suggest the potential role of NEP in cleaving the abundant Aβ in AD. However, the underlying mechanism for the reduction of NEP in AD is not fully elucidated. To distinguish between primary decreases in NEP activity that might contribute to Aβ accumulation and decreases secondary to neurodegenerative changes in AD, NEP levels and activity were significantly increased in AD stages but negatively with age in AD patients, suggesting that reduction in NEP activity is not the primary cause of Aβ accumulation in AD, but rather a late‐stage phenomenon secondary to neurodegeneration.^37^

In contrast to the potential benefits of enhanced Aβ clearance, sustained NEP activation may be detrimental because NEP can degrade a wide range of circulating peptides. Although transgenic mice expressing high levels of human NEP do not show detectable adverse effects, potential side effects of a chronic increase in NEP activity have not been fully established.^38^ Transgenic overexpression of human NEP in neurons shortened the life span of flies and caused age‐dependent axon degeneration in the brain.^27^ Moreover, NEP overexpression in neurons decreased CREB‐mediated transcription in the fly, and reducing CREB activity in neurons was sufficient to cause premature death.^27^ These data suggest that a reduction in cyclic AMP response element (CRE)‐binding protein (CREB)‐mediated transcription underlies premature death induced by NEP overexpression. However, premature death and neurodegeneration induced by NEP expression may be because of a consequence of aberrant expression in brain regions that do not usually express NEP, and enhancement of endogenous NEP may not cause these effects.^27^ Taken together, these data suggest both the protective and detrimental effects of NEP activity on AD neuropathology (Table 1).

**TABLE 1:** Role of NEP in AD neuropathology.

Study type	Findings	Ref.
Experimental study	Increasing expression of the NEP gene attenuates Aβ level in the hippocampus of NEP deficient mice	Iwata et al.^24^
Experimental study	Neprilysin gene transfer reduces human amyloid pathology in transgenic mice	Marr et al.^25^
In vitro study	Human NEP is capable of degrading Aβ peptide not only in the monomeric form but also in the pathological oligomeric form	Kanemitsu et al.^26^
Experimental study	Intra‐neuronal Aβ42 deposit is highly cleaved by NEP in mice	Iijima‐Ando et al.^27^
Experimental study	A deficiency of NEP accelerates the formation of extracellular Aβ deposits, Aβ angiopathy, synaptic dysfunctions and memory deficits in transgenic mice	Huang et al.^28^
Experimental study	Delivery of NEP to the brains of APP transgenic mice reduces extracellular Aβ deposits, synaptic dysfunction and premature death	Hemming et al.^29^
Experimental study	Higher expression of NEP is associated with a lower incidence of spatial memory deficit and development of AD in mice	Poirier et al.^30^
Clinical study	In patients with late‐onset AD, NEP mRNA and protein levels are selectively reduced in brain regions highly vulnerable to AD pathology	Yasojima et al.^39^
Post‐mortem study	Reduction of NEP levels contributes to the onset and/or progression of late‐onset AD	Hellstrom‐Lindahl et al.^32^
A cohort study	Overexpression of NEP can decrease the risk of AD by reducing intra‐neuronal Aβ accumulation in patients following traumatic brain injury	Johnson et al.^34^
A case–control study	NEP activity is reduced in the CSF of the patients with cognitive impairments	Grimmer et al.^36^
Post‐mortem study	NEP is not the primary cause of Aβ accumulation in AD, but secondary to neurodegeneration	Miners et al.^37^

Abbreviations: Aβ, amyloid beta; AD, Alzheimer's disease; APP, amyloid precursor protein; NEP, neprilysin.

## 3 NEP INHIBITORS IN AD

**FIGURE 3:**
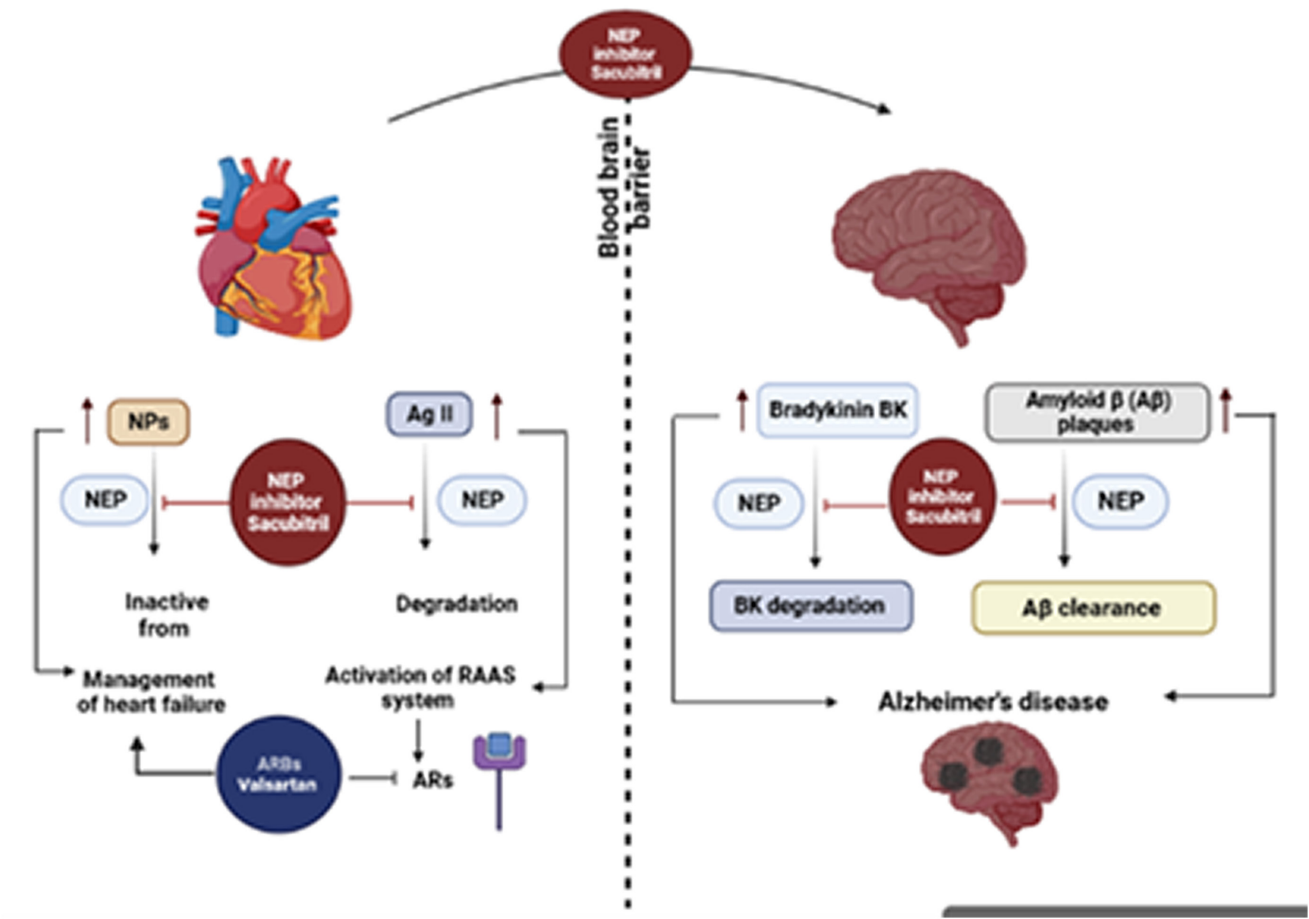
LCZ696 use and the risk of AD: Angiotensin receptor blocker (ARB) valsartan inhibits angiotensin receptors (ARs) which are activated by the renin‐angiotensin‐aldosterone system (RAAS). Besides, NEP inhibitor sacubitril increases angiotensin II (AngII) and natriuretic peptides (NPs). ARNI can cross blood–brain barrier (BBB) and inhibit central NEP leading to a reduction of Aβ clearance and bradykinin (BK) degradation with the development of AD.

NEP inhibitors are rarely used alone but commonly used in combination with angiotensin II (AngII) receptor blockers (ARBs) in the management of heart failure through modulation of RAS and expression of natriuretic peptides (NPs). Sacubitril was the first NEP inhibitor approved in 2015 to manage heart failure.^40^ Sacubitril, combined with valsartan, was initially named LCZ696, an angiotensin receptor blocker and NEP inhibitor angiotensin receptor neprilysin inhibitor (ARNI). LCZ696 decreases blood pressure more than valsartan alone in hypertensive patients.^41^ In a PARADIGM‐heart failure study, LCZ696 was more effective than angiotensin‐converting enzyme inhibitor (ACEI) enalapril in the management of heart failure.^42^ The maximal concentration of the valsartan component of LCZ696 is reached in 1.7–2.2 h and 0.5–1.1 h for AHU377 with the active metabolite LBQ657. LBQ657 exerts its inhibitory effect on NEP leading to an observed increase in both atrial NPs and guanosine monophosphate (cGMP). A dose escalation study in 83 healthy participants showed a maximal 40% increase in mean cGMP levels at 4 h and significant increases at 12 h post‐dose with a return to baseline levels at 24 h after administration of LCZ696.^40^ Peak concentrations of LBQ657 and valsartan were reached within a similar time frame demonstrating comparable pharmacokinetic properties.^41^ This is in contrast with NEP inhibitor omapatrilat which exerted delayed NEP inhibition when compared with ACE inhibition.^43^ Regarding the ability of NEP inhibitors to cross BBB, both NEP inhibitors thiorphan and candoxatrilate cannot cross BBB, whereas acetyl‐thiorphan and AHU‐377 can cross BBB efficiently.^44^ Notably, BBB dysfunction is linked with Aβ‐induced CAA in AD.^45^ Of note, NEP inhibitors can cross BBB and affect Aβ metabolism. NEP inhibitor LBQ657 (which is rapidly metabolized into the active NEP inhibitor LBQ657 by enzymatic cleavage of its ethyl ester) can cross the BBB and increases CSF Aβ in cynomolgus monkeys and Aβ‐_38_ in human CSF but does not increase Aβ levels in the brain tissue of monkeys.^46^ Evaluation of the ability of NEP inhibitors to cross BBB in patients with mild cognitive dysfunction and pre‐AD is complex. Consequently, NEP inhibitors can cross dysfunctional BBB in AD, causing severe deteriorations in cognitive functions.^43^ Prolonged use of NEP inhibitors may increase the risk of AD development due to inhibition of the activity of NEP which is involved in the cleavage, degradation and clearance of Aβ (Figure 3).

### 3.1 Preclinical findings

Preclinical studies established that using NEP inhibitors might induce AD‐like diseases in animals.^44^ The use of NEP inhibitors in mice aggravates AD development.^44^ NEP deficiency resulted in defects both in the degradation of exogenously administered Aβ and in the metabolic suppression of the endogenous Aβ levels. The regional levels of Aβ in the NEP‐deficient mouse brain were in the distinct order of hippocampus, cortex, thalamus/striatum and cerebellum, where the hippocampus has the highest level and cerebellum the lowest, correlating with the vulnerability to Aβ deposition in brains of humans with AD.^43^, ^44^ These observations propose that even partial downregulation of NEP activity can participate in AD development by promoting Aβ accumulation. Thus, NEP inhibitors can hasten the progression of AD neuropathology via inhibition of the Aβ clearance pathway.^44^ Furthermore, an experimental study illustrated that LCZ696 increased Aβ concentration in the CSF in animals but not in the brain. Conceivably, the potential adverse effects of sacubitril on Aβ degradation might be counterbalanced by ARB's beneficial vascular effects.^43^ LCZ696 may compromise Aβ peptide degradation in the brain, and may thus accelerate AD progression.^47^ Long‐term effects of NEP inhibitors may promote AD development via augmentation of Aβ accumulation and related CAA.^41^ Besides, NEP inhibitors increase the risk of developing late‐onset axonal polyneuropathy.^41^ An updated experimental study demonstrated that LCZ696 increases AD risk compared with valsartan alone in rats.^48^ Valsartan can decrease the risk of AD development as it selectively blocks AT_1_R as RAS inhibitors have been associated with the reduction of brain damage in different experimental and clinical models of neurodegenerative diseases.^49^ In addition, LCZ696 extravagant colchicine‐induced cognitive impairment in rats by increasing Aβ accumulation, oxidative stress and inflammation compared to valsartan alone.^50^ Hence, LCZ696 triggers a deleterious effect on cognitive impairment in the colchicine‐induced AD rat model. Henceforth, special caution should be taken following long‐term intake of LCZ696 on cognitive functions. These findings come from animal model studies, though they may not apply to humans. Indeed, NEP polymorphism is associated with the development of CAA and AD. Remarkably, LCZ696 has a neuroprotective effect by inhibiting homocysteine‐induced BBB injury both in vitro and in vivo.^51^ As well, the neuroprotective effect of LCZ696 is also mediated by suppressing expression of pro‐inflammatory cytokines such as IL‐6.^51^ These observations need to be meticulously tested in clinical trials. If confirmed in human subjects, this would be of much superior concern in hypertensive patients who are treated for years and even decades than in heart failure where survival is reduced.

### 3.2 Clinical findings

It has been shown that using NEP inhibitors may increase the risk of AD by conciliation of Aβ clearance with induction of Aβ‐induced cerebral angiopathy in high‐risk patients.^43^ Long‐term use of NEP inhibitors in patients with NEP polymorphism results in a more severe reduction of neuronal NEP activity and higher Aβ accumulation.^52^ Thus, NEP polymorphism is regarded as a genotype risk factor for the development of AD.^35^ It has been shown that younger patients receiving LCZ696 have the potential for longer‐term exposure and consequent increased risk of early‐onset AD described in subjects less than 65 years of age and has a more rapid progression than the typical late‐onset AD.^53^ In the 36‐week PARAMOUNT study, no excess in neurological adverse effects was seen.^54^ However, these trials were not prospectively designed to specifically evaluate the effects of LCZ696 on neurocognitive function, AD, or other forms of dementia. These findings implicate LCZ696 in the pathogenesis of AD.

Conversely, many clinical studies and trials established that long‐term use of LCZ696 did not affect cognitive functions and did not increase AD risk.^42^, ^55^ McMurray et al.^55^ indicated that dementia‐related adverse effects were not increased in the arm treated with LCZ696 and confirmed that serial cognitive tests will be performed in the PARAGON‐HF trial; this latter point is critical as the evaluation of the cognitive function was not defined in the original PARADIGM‐HF article.^42^ In addition, the administration of LCZ696 for 2 weeks in humans did not affect Aβ concentration in the CSF.^47^ These observations suggest that short‐term therapy with NEP inhibitors is safe. Cognitive impairment and dementia‐related adverse events were also not associated with the use of LCZ696.^55^ Nonetheless, the duration of trials regarding the potential effect of LCZ696 on cognitive function might be short; thus, long‐term follow‐up for the significant effect of LCZ696 is advisable. A systematic review and meta‐analysis of randomized controlled clinical trials demonstrated that LCZ696 had no serious adverse effects compared to placebo in hypertensive patients.^56^ LCZ696 was more effective in reducing blood pressure and had a higher rate of blood pressure control compared with ARBs. LCZ696 had no difference in the incidence of adverse events or serious adverse events compared to ARBs^56^ suggesting that LCZ696 has a greater antihypertensive efficacy and an equal tolerability profile. Despite theoretical concerns regarding the incidence of neurocognitive impairments with the use of LCZ696, a large cohort study showed that neurocognition in patients with heart failure on LCZ696 did not differ from that on ACEIs or ARBs.^57^ The potential exists that treatment of LCZ696 through the inhibition of NEP by LBQ657 may result in accumulation of Aβ. In a double‐blind, randomized, parallel‐group, placebo‐controlled study, healthy subjects received once daily LCZ696 (400 mg, *n* = 21) or placebo (*n* = 22) for 14 days showed that LCZ696 did not cause changes in CSF levels of Aβ_1‐42_ compared with placebo, despite achieving CSF concentrations of LBQ657 sufficient to inhibit NEP.^58^ However, CSF Aβ_1–38_ was increased in subjects treated with LCZ696 compared with placebo was observed. Nevertheless, there was no apparent relationship between CSF Aβ_1–38_ concentrations and LBQ657 plasma and CSF concentrations. Aβ_1–38_ is soluble, more readily transported within the brain interstitial space and into the CSF, and may be more susceptible to increase with NEP inhibition compared with the more hydrophobic and aggregation‐prone isoform Aβ_1–42_.^58^ A total of 6 randomized trials with 11,821 subjects included in meta‐ analysis showed no any risk for the development of cognitive impairment in patients on LCZ696 treatment.^58^ A recent clinical trial on 592 patients from 137 centres in 20 countries were randomized 1:1 to either LCZ696 (target dose 97/103 mg twice daily) or valsartan (target dose 160 mg twice daily) showed no evidence that NEP inhibition increased the risk of cognitive impairment in patients with heart failure.^59^ A retrospective cohort study revealed that patients with heart failure treated by LCZ696 for 3 months did not experience cognitive impairments.^60^ Cannon et al.^61^ analysed the dementia‐related adverse effects in the PARADIGM‐HF trial and found no evidence that LCZ696, compared with enalapril, increased dementia‐related adverse events. A retrospective cohort study involved 858 patients with heart failure on LCZ696, and 1209 patients on ACEIs or ARBs followed for 3 years revealed that LCZ696 was more effective than ACEIs or ARBs in alleviating neurocognitive impairments associated with heart failure.^62^ The concern about increased cerebral Aβ deposition with LCZ696 was always hypothetical and multiple enzymatic and other Aβ clearance pathways exist in the brain that would likely compensate for any decreased clearance related to NEP inhibition. The absence of any negative effect on cognitive function is very important in removing a concern some doctors had about long‐term treatment with LCZ696.

**FIGURE 4:**
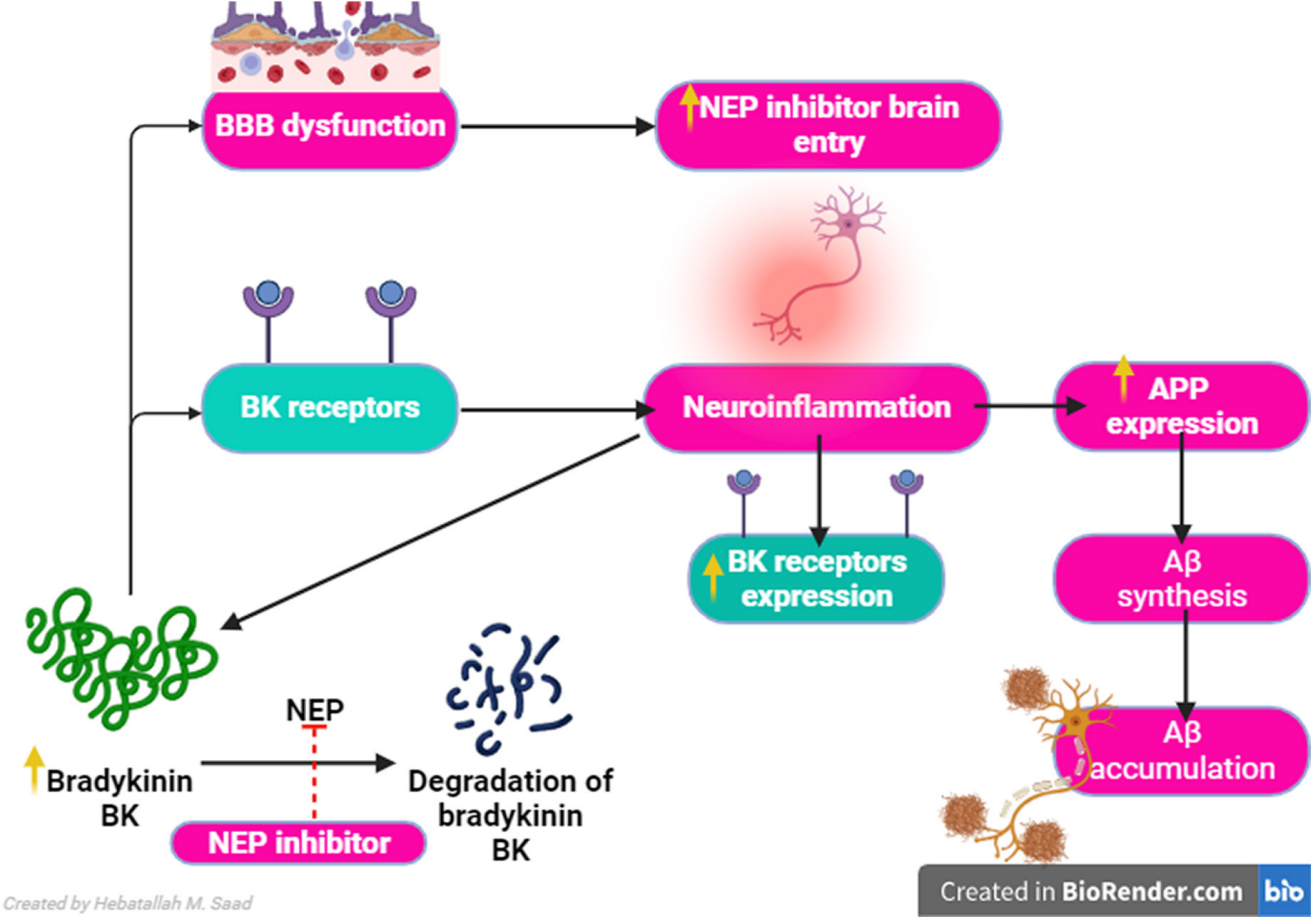
Possible mechanism of neprilysin (NEP) inhibitors‐induced AD: NEP inhibitors inhibit bradykinin (BK) degradation leading to an increase in BK level which through activation of BK receptors leads to neuroinflammation. In turn, neuroinflammation increases the expression of BK receptors and amyloid precursor protein (APP) which enhances the synthesis and accumulation of Aβ. In addition, BK causes blood–brain barrier (BBB) dysfunction and increases entry of NEP inhibitors into the brain.

Furthermore, different studies have revealed promising safety findings regarding the use of LCZ696; those studies were highly criticized for the targeted population and short monitoring time.^63^ The underlying mechanism of LCZ696‐induced AD is related to the induction expression of bradykinin (BK) which triggers BBB dysfunction leading to more passage of LCZ696.^64^ Besides, BK through activation of the BK2 receptor (B2R), promotes the progression of neuroinflammation and accelerates AD development. BK through expression induction of APP increases production Aβ with further accumulation and deposition. Together, NEP inhibitors through inhibition of NEP with overexpression of BK trigger Aβ accumulation (Figure 4).^64^

Together and till now, there is no solid evidence that NEP inhibitors adversely affect cognitive function despite evidence from experimental and preclinical studies for the association between NEP inhibitor use and the risk of AD (Table 2).

**TABLE 2:** Role of NEP inhibitors in AD neuropathology.

Study type	Findings	Ref.
Experimental study	NEP inhibitors trigger AD‐like diseases in mice	Hüttenrauch et al.^44^
Experimental study	LCZ696 increased Aβ concentration in the CSF but not in the brain	Bavishi et al.^43^
Experimental study	LCZ696 accelerates AD progression in animals	Vodovar et al.^47^
Experimental study	Long‐term effects of NEP inhibitors promote AD and late‐onset axonal polyneuropathy	Auer‐Grumbach et al.^41^
Experimental study	LCZ696 increases AD risk compared with valsartan alone in rats	El‐din Hussein et al.^48^
Experimental study	LCZ696 accelerates colchicine‐induced cognitive impairment in rats	Hammadi El‐din Hussein et al.^50^
Preclinical studies	LCZ696 has a neuroprotective effect by inhibiting homocysteine‐induced BBB injury	Li et al.^51^
Observational study	Long‐term use of NEP inhibitors in patients with NEP polymorphism results in severe Aβ accumulation	Krittanawong et al.^52^
A cohort study	Longer‐term use of LCZ696 increases the risk of early‐onset AD in subjects less than 65 years	Stanley et al.^53^
A phase 2 double‐blind randomized controlled trial	In the 36‐week PARAMOUNT study, no excess in neurological adverse effects was seen	Solomon et al.^54^
Clinical trial	Dementia‐related adverse effects were not increased in the arm treated with LCZ696	McMurray et al.^46^
Clinical trial	PARADIGM‐HF trial found no evidence that LCZ696 increased dementia‐related adverse events compared with enalapril	Cannon et al.^61^
A retrospective cohort study	LCZ696 was more effective than ACEIs or ARBs in alleviating neurocognitive impairments in patients with heart failure	Grewal et al.^62^

Abbreviations: Aβ, amyloid beta; ACEIs, angiotensin‐converting enzyme inhibitors; AD, Alzheimer's disease; APP, amyloid precursor protein; ARBs, Angiotensin receptor blockers; NEP, neprilysin.

## 4 MECHANISTIC ROLE OF NEP INHIBITORS IN AD

### 4.1 NEP inhibitors and hyperglycaemia

NEP is involved in the cleavage and degradation of more than thirty peptides, mainly glucagon‐like peptide 1 (GLP‐1), neuropeptide Y (NPY), insulin, substance P, β endorphin and encephalin.^65^ NEP inhibition increases various peptides involved in inflammation, glucose homeostasis and nerve conduction.^65^ Inhibition of NEP leads to beneficial systemic effects by regulating insulin sensitivity, lipid metabolism, satiety and pancreatic β cell function.^66^, ^67^, ^68^, ^69^, ^70^

NEP inhibitors could effectively be used in the management of Type 2 diabetes mellitus (T2DM).^65^, ^71^

Regulation of blood glucose by NEP inhibitors in T2DM patients may prevent hyperglycaemia‐induced AD.^65^ In vitro study demonstrated that rat embryonic cortical neurons subjected to high glucose concentration results in apoptosis and cell deaths. Also, glucose triggers tau protein phosphorylation and Aβ accumulation in brain tissues.^72^ Besides, an experimental study revealed that diabetic mice have a higher capacity for increased tau protein phosphorylation.^72^ These in vitro and in vivo findings suggest a possible association between T2DM and the risk of AD.

It has been reported that T2DM patients have a 50%–75% risk of AD development, and AD patients have a higher risk of developing T2DM.^72^, ^73^ Data from clinical studies support the use of NEP inhibitors in the prevention and treatment of T2DM.^74^, ^75^ A prospective study involving 73 patients with heart failure, of which 16 patients had T2DM, changed the therapeutic strategy from ARBs or ACEIs to ARNI for 3 months, led to a significant reduction of NEP levels with a significant decrease in fructosamine levels, a glycated biomarker protein in T2DM.^75^

One important mechanism by which NEP inhibitors improve blood glucose homeostasis is by increasing circulating GLP‐1.^76^ NEP inhibitors control blood glucose and prevent the development of T2DM.^77^, ^78^ An in vitro study confirmed that NEP inhibitors enhance the release of GLP‐1 and insulin^78^; thus, inhibition of NEP in the pancreatic β cell could be a possible mechanism for glucose haemostasis. Notably, high‐fat diet‐induced obesity in NEP‐deficient mice produces a less harmful effect on insulin sensitivity due to high circulating GLP‐1 levels.^79^ In addition, NEP increases the expression of dipeptidyl peptidase 4 (DPP4), causing a reduction in insulin and GLP‐1 levels.^80^ Thus, NEP inhibitors may inhibit DPP4 expression, ameliorating insulin and GLP‐1 levels.^79^ Also, BK improves blood glucose by increasing insulin sensitivity.^81^ BK which is degraded by NEP is increased following the use of NEP inhibitors. Therefore, NEP inhibitors could be effective in attenuation of hyperglycaemia‐induced AD. In this bargain, increasing GLP‐1, BK and inhibiting DPP4 expression by using NEP inhibitors may positively affect the management of AD pathogenesis.

### 4.2 NEP inhibitors and GLP‐1

It has been shown that GLP‐1 and long‐acting GLP‐1 analogues are not only degraded by DPP4 but also by NEP.^82^, ^83^ NEP inhibition by specific inhibitors augments circulating GLP‐1, which has a neuroprotective effect against Aβ‐induced cytotoxicity, neuroinflammation, synaptic dysfunction‐induced memory and cognitive dysfunctions.^82^ Long^84^‐acting GLP‐1 analogues are highly resistant to the effect of DPP4.^85^ Notably, long‐acting GLP‐1 analogues are mainly degraded by NEP, whereas endogenous GLP‐1 is mainly degraded by DPP4.^85^ Endogenous GLP‐1 plays a critical role in regulating cognition and neuronal growth. Endogenous GLP‐1 crosses BBB and inhibits brain oxidative stress and neuroinflammation.^86^ A study revealed that GLP‐1 deficient mice are highly susceptible to neuronal loss and the development of neurodegenerative disorders.^87^ Additionally, increasing GLP‐1 receptor signalling attenuates infarct size and neuroinflammation.^88^ Experimental studies confirmed that GLP‐1 and GLP‐1 analogues attenuate Aβ accumulation, tau protein phosphorylation, neurotoxicity and neuroinflammation.^89^, ^90^ The inhibition of GLP‐1 degradation is linked with cognitive enhancement and reduced AD development risk.^91^ A prospective study involving 253 T2DM patients on sitagliptin or non‐sitagliptin treatments illustrated that DPP4 inhibitor sitagliptin improved cognitive function compared to other diabetic therapies.^91^ The cognitive enhancement effect of DPP4 inhibitors is related to the augmentation of GLP‐1.^84^ Despite the risk of Aβ accumulation in NEP‐deficient mice, they experience less cognitive dysfunction due to the high concentration of GLP‐1 in the brain.^92^ NEP‐deficient aged mice surprisingly had a higher neurocognitive profile and long‐term potentiation.^92^ This effect might vary due to different expressions of neuronal GLP‐1. GLP‐1 is released from the brainstem, mainly from tractus solitaries, and consequently transported to other brain regions.^93^ Neuronal GLP‐1 regulates insulin sensitivity and glucose homeostasis in the brain,^93^ so it may play a role in the mitigation of brain insulin resistance (IR), a hallmark of AD.^94^, ^95^ Emerging evidence from animal and human studies highlighted that insulin promotes synaptogenesis, cerebral biogenetics, and the turnover of neurotransmitters.^94^ Also, insulin prevents tau protein phosphorylation and enhances Aβ clearance with modulation of inflammation, vasoreactivity and lipid metabolism.^94^, ^96^ The defect in insulin receptor substrate 1 (IRS‐1) due to the Aβ‐induced release of pro‐inflammatory cytokines is responsible for developing brain IR in AD.^97^ Thus, restoring brain insulin sensitivity by augmenting GLP‐1 and inhibiting neuronal NEP could be a therapeutic strategy in treating AD.

Furthermore, there is an association between the failure of brain GLP‐1 and the development of AD.^98^ Animal and human studies illustrated that GLP‐1 analogues like liraglutide could reverse brain IR in AD.^97^ Vargas‐Sori et al.^99^ clarified that liraglutide is crucial in counteracting abnormal brain metabolism and associated inflammatory disorders. Likewise, liraglutide prevents tau phosphorylation, neuronal injury and synaptic dysfunction with improved cognitive function in animals.^99^ However, the primary effect of liraglutide in AD patients is limited, and ongoing clinical trials regarding the effect of liraglutide on AD patients may provide more conclusive findings.

**FIGURE 5:**
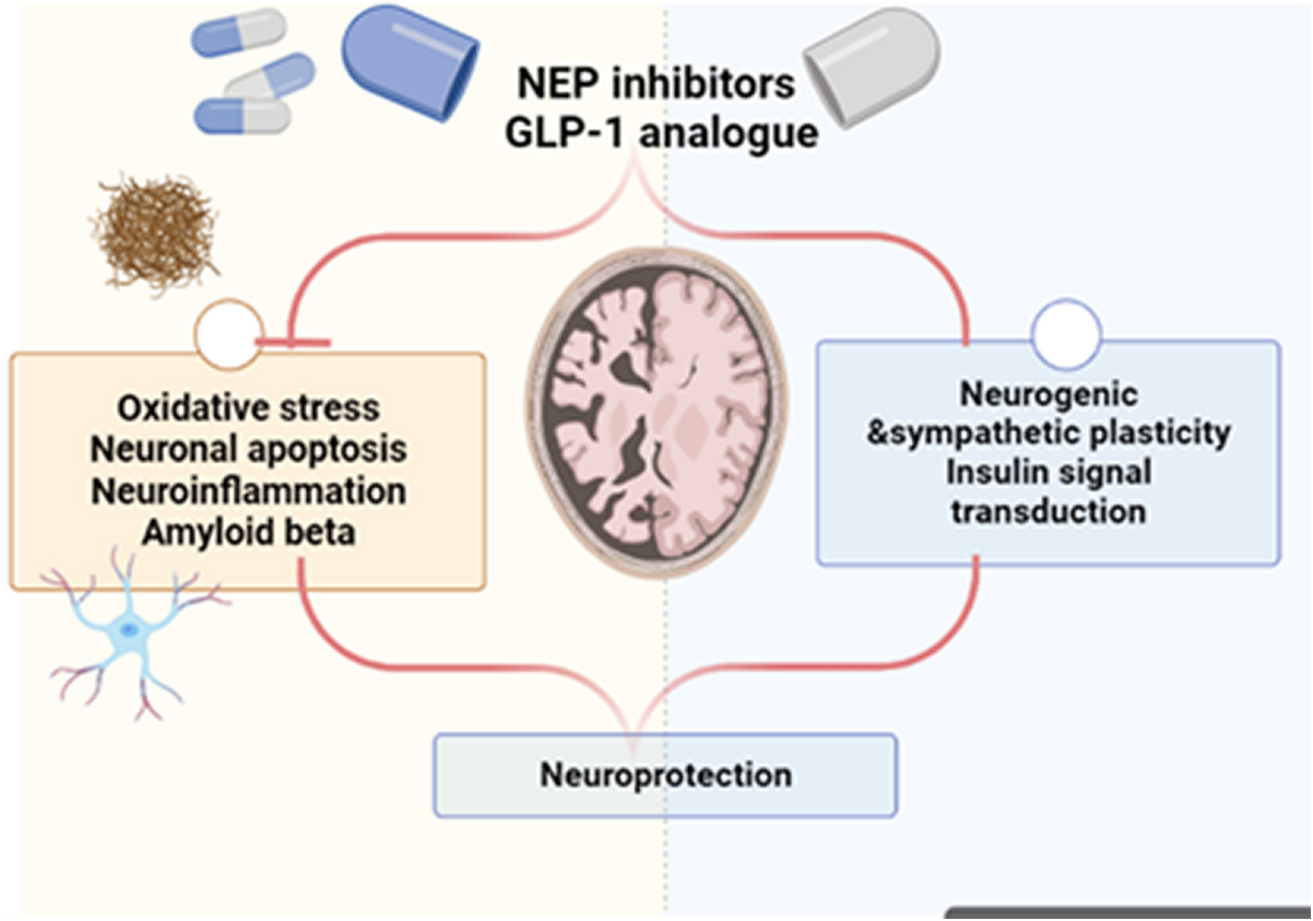
NEP inhibitors and GLP‐1 in AD.

These findings suggest that augmentation of brain GLP‐1 by NEP inhibitors may prevent AD development through modulation of brain glucose homeostasis, attenuation of brain IR and mitigation of neuroinflammation and oxidative stress (Figure 5).

### 4.3 NEP inhibitors and substance P

Substance P is a neurotransmitter, neurotrophic, and neuromodulator that regulate various functions in the CNS.^100^ It has neuroprotective effects in brain tissues by activating the non‐amyloidogenic process, which decreases the generation of toxic Aβ through the APP amyloidogenic pathway.^101^ The neuroprotective effect of substance P is mediated by the activation of voltage‐gated K channel current, which is inhibited in AD. Thus, the inhibition of substance P plays a critical role in the pathogenesis of AD.^101^ AD animal model studies showed reduced substance P levels in the CSF and brain.^101^ NEP inhibitors increase substance P levels by inhibiting their degradation process. Previous post‐mortem studies indicated that NEP activity was altered, leading to an increased half‐life of substance P, which increases NEP expression in senile dementia.^102^ However, expression of substance P is reduced in different brain regions but increased in the thalamus and hippocampus in AD.^103^ Substance P level is reduced in early‐onset AD and increased in late‐onset AD.^104^ A post‐mortem study revealed that CSF substance P level was mainly increased in late‐onset AD compared to controls.^104^ Increasing CSF substance P in late‐onset AD might be a compensatory mechanism to mitigate neuroinflammation. However, substance P level in the brain is positively correlated with Aβ accumulation in AD patients.^105^ This effect might be due to the alteration of NEP expression in the temporal cortex.^102^ Activation of voltage‐gated K channel current by substance P prevents the toxic effect of Aβ on this channel which exerts a neuroprotective effect.^106^ Systemic administration of substance P 50 μg/kg for 7 days inhibits the deleterious effect of Aβ on the cognitive function in rats with AD.^106^ Substance P attenuates Aβ‐induced neuronal apoptosis through modulation of voltage‐gated K channel current.^107^ Of interest, Aβ accumulation induces a reduction in the expression of substance P in the hippocampus.^108^

In silico study conducted by Satarker et al.^109^ demonstrated that substance P could be effective in AD through the modulation of neuroinflammation. However, this study was not confirmed by clinical evidence. These findings suggest that the reduction of brain substance P is associated with AD neuropathogenesis. Therefore, augmentation of substance P by NEP inhibitors could effectively manage AD.

### 4.4 NEP inhibitors and NPY

NPY is a neuropeptide that regulates multiple physiological functions centrally and peripherally.^110^, ^111^ NPY is the most abundant neuropeptide in the CNS; it is secreted along with other neurotransmitters, such as glutamate and GABA.^110^ NPY is released mainly from sympathetic neurons and the hypothalamus to regulate different functions, including feeding, stress, blood pressure and cardiac rhythm.^112^

Regarding the relation between NPY and AD, previous studies revealed that expression levels of NPY were reduced in AD animals and humans.^113^, ^114^ Post‐mortem study showed that NPY level, mainly in cortical neurons, was lower in AD patients' brain tissues than in controls.^113^ Loss of immunoreactivity to NPY in temporal and frontal lobes was reduced by 95% in AD patients due to progressive neuronal loss. Alom et al.^114^ confirmed that CSF NPY level in AD patients was lower compared to healthy controls. A case–control study involving 25 AD patients and 25 healthy controls illustrated that plasma NPY levels were lower in AD than in controls.^115^ A recent review by Pain et al.^116^ found that increasing of NPY levels in plasma and CSF of neurodegenerative patients reflects a counterbalance mechanism to reduce neuroinflammation and neuronal injury. NPY acts as an anti‐inflammatory and anti‐apoptotic neuropeptide that halt the progression of neurodegeneration and AD neuropathology.^116^

Moreover, immunoreactivity to the NPY is reduced in hippocampal interneurons in the pre‐symptomatic AD model in mice.^117^ Similarly, the neuroprotective effect of NPY in AD is mediated through the inhibition of Aβ‐induced oxidative stress and lipid peroxidation.^118^ Croce et al.^119^ revealed that pretreatment with NPY attenuates Aβ‐induced neurotoxicity through increasing nerve growth factor and brain‐derived neurotrophic factor (BDNF). Remarkably, the degradation of NPY by NEP produces C‐terminal fragments, which also have neuroprotective effects in AD.^119^ Excessive Aβ accumulation in the hypothalamus disrupts NPY signalling leading to a reduction in feeding drive with subsequent weight loss in the late stage of AD.^118^ Therefore, NEP inhibitors may improve AD pathology by increasing the level of NPY, which has a direct neuroprotective effect, or through augmentation of BDNF.

### 4.5 NEP inhibitors and bradykinin

BK is a peptide chain consisting of nine amino acids that promote inflammation and vasodilation by increasing the release of nitric oxide (NO), prostaglandin and endothelium‐derived hyperpolarizing factor.^120^, ^121^, ^122^ BK and its metabolites have pro‐inflammatory effects via activation of B1R and B2R.^120^ ACE and NEP metabolize BK; thus, ACEIs and NEP inhibitors increase BK levels, causing angioedema.^123^ Despite increasing BK and NPs by NEP inhibitors, these agents have modest effects in managing heart failure and hypertension and increasing brain BK affects cognitive functions.^123^ For example, a previous experimental study showed that infusion of BK into the brain, mainly the hippocampus, led to AD‐like disease in rats characterized by memory deficit and learning dysfunction through induction of tau protein phosphorylation.^124^ Thus, B1R antagonists attenuate Aβ deposition in AD mice models.^125^ In addition, B1R antagonists reduce the development of BBB dysfunction in mice with experimental stroke.^126^ Remarkably, expression of B1R in astrocytes is increased around Aβ plaques in mice,^127^ while BK expression in the CSF is augmented following injection of Aβ in mice.^128^ Of interest, BK is involved in the activation processing of APP to Aβ.^126^, ^127^ These observations suggest that BK is implicated in the pathogenesis of AD and associated inflammation. The conclusions of these studies are based on experimental studies and not human ones.

Notoriously, Strickland et al. illustrated that the contact system in AD is highly deregulated, causing abnormal BK expression.^129^ However, the relationship between BK and AD pathogenesis in humans is limited. A recent post‐mortem study observed that CSF BK level was higher in AD patients compared to healthy controls.^130^ Therefore, increasing BK levels in AD patients may lead to inflammation‐induced cognitive impairment and AD progression.^130^

It has been shown that BK can induce BBB dysfunction, a hallmark of AD, even before the development of brain atrophy.^45^ BBB injury facilitates the entry of neurotoxic‐derived blood products linked with systemic inflammation.^45^, ^131^ Of note, systemic inflammation is linked with higher expression of BK, whereas systemic inflammation and higher BK can initiate BBB injury and neuroinflammation.^45^ BK‐induced BBB injury and neuroinflammation could be related to the upregulation of NO expression.^132^ Therefore, higher BK expression in AD is associated with cognitive deficits and memory disorders. However, not all AD patients have higher CSF BK levels.^130^ The underlying mechanism of BK's role in AD is related to microglial activation and neuroinflammation.^133^ Increased kallikrein enzyme expression in the AD brain is associated with higher BK in the brain parenchyma.^134^ Cerebral infusion of Aβ_1‐40_ promotes kallikrein enzyme activation in the frontal and temporal cortex with subsequent BK release in dementia and AD.^134^ Thus, elevated BK release contributes to the induction of cerebral vascular permeability and impairment of cerebral blood flow in AD.^134^ Despite these associations, many AD hypertensive patients treated with ACEIs had no significant changes in BK levels compared to controls.^130^ Analysis and exclusion of cofounder factors in a cohort study of AD patients revealed a significant association between cognitive dysfunction and BK level in AD.^130^

Higher expression of BK in AD is not fully understood, though it may relate to higher expression of degrading enzymes like ACE and NEP in AD patients. ACE had been reported to be dysregulated in AD patients as its plasma levels were reduced while its CSF levels were increased.^135^, ^136^ It has been observed that ACEIs improve memory function in AD despite increased plasma BK levels.^137^ Another study revealed that ACEIs might increase the risk of AD through inhibition of ACE‐mediated conversion Aβ_1‐42_ to Aβ_1‐40._ ^138^ Remarkably, hyperfibrinolysis induces BBB injury through the generation of endogenous BK.^139^ Besides, the overexpression of tissue plasminogen activator (tPA) and plasmin are linked with AD development by accelerating the accumulation of Aβ.^140^, ^141^ Therefore, higher BK in AD could be related to hyperfibrinolysis and higher tPA.

These findings highlighted a potential controversy regarding the possible role of BK in AD. In this state, NEP inhibitors may adversely affect the neurocognitive function in AD through increasing BK levels. Notably, NEP inhibitors may increase the risk of AD progression by increasing BK and reducing Aβ degradation.^41^ These findings were primarily related to animal studies and were not confirmed in human studies. Therefore, the detrimental effects of NEP inhibitors related to AD risk through BK need to be elucidated in clinical studies.

### 4.6 NEP inhibitors and natriuretic peptides

NPs are a group of peptides released from cardiomyocytes in response to volume expansion and cardiac wall stretch. NPs regulate body homeostasis through natriuresis, vasodilation and diuresis.^142^, ^143^ There are three types of NPs, including atrial natriuretic peptide (ANP), C‐type natriuretic peptide (CNP) and brain natriuretic peptide (BNP).^1^ NPs act on specific natriuretic peptide receptors (NPR), including NPR‐A, NPR‐B and NPR‐C.^140^, ^143^ NPs are also expressed in the brain, mainly in neurons, glial cells and neurovascular units.^8^ They improve BBB integrity, synaptic plasticity, memory function, neurotransmitter release and attenuation of neuroinflammation.^8^ Notably, a potential link between NPs and AD development is mainly related to cardiovascular complications.^5^ A 5‐year population‐based cohort study involving 464 individuals aged >75 showed that BNP was an independent risk factor associated with the development of AD.^9^ According to general result that the reduction of neuroprotective NPs is associated with AD development though BNP augments AD.^5^ In addition, higher NPs plasma is connected with cognitive dysfunction, and a higher NPs level in the CSF is associated with a low Aβ level in the CSF.^10^ Thus, NPs could be a possible diagnostic biomarker in AD. Higher expression of NPR‐A is associated with AD neuropathology.^11^ NPs may be potential diagnostic and/or therapeutic markers for AD. Decreased action of NP in the brain might impair the structural and/or functional integrity of the brain and predispose individuals to a higher risk of cognitive decline.^11^ Levels of BNP in the CSF of AD patients, coupled with higher amounts of NPR‐A in the brain tissue of AD patients. This may suggest an impaired function of NP in the brain of AD patients, which could, in turn, accelerate neuroinflammation, oxidative stress and neurodegeneration. Such alteration may ultimately hamper the functional and/or structural integrity of the brain and predispose individuals to a higher risk of cognitive decline. One explanation for reduced levels of BNP in the CSF could be attributed to their elevated levels in the systemic circulation.^11^ In fact, systemic and central NP might act in a feedback loop such that increased NP in the plasma inhibits the production and/or biological activity of NP in the brain.^11^ The systemic effect of high NPs levels is linked with cognitive decline independent of cardiovascular risk factors due to negative feedback inhibition.^12^ Also, higher systemic NPs levels block hypothalamic NPs signalling.^144^ Interestingly, a post‐mortem AD patient is associated with low BNP in the CSF^8^; thus, the reduction of brain BNP may accelerate the development of neuroinflammation, neurodegeneration, oxidative stress and BBB dysfunction.^145^ Notoriously, NPs levels and NPR expression are higher in the hypothalamus and hippocampus of healthy humans, suggesting a neuroprotective role of NPs.^8^ These findings suggest that the reduction of neuroprotective NPs is associated with AD development. Thus, NPs agonists or NEP inhibitors may improve cognitive function and reduce AD pathogenesis by targeting the NPs system.

It has been reported that ARNI increases of NPs levels by inhibiting NEP, which is involved in the degradation of NPs. A study involving 23 patients with heart failure treated with ARNI showed that the ANP level was sustainably increased. In contrast, BNP was not increased without changing CNP levels in mice^146^; these findings proposed that the ARNI effect mainly increases ANP. Concerning Aβ, ANP competes with Aβ for clearance across BBB.^147^ Thus, increasing the level of ANP by ARNIs may inhibit Aβ clearance. A meta‐analysis and systematic review illustrated that mid‐regional (MR) pro‐ANP has predictive value in the conversion and progression of pre‐dementia to clinical AD.^148^ However, ARNI reduces pro‐ANP levels.^146^ The conflicting evidence concerning the role of NEP inhibitors in the progression of AD through the NPs signalling system is not confirmed clinically. Therefore, additional preclinical and clinical studies are warranted in this regard.

These verdicts proposed that NEP inhibitors have a protective effect against AD development through increasing GLP‐1, NPY and substance P. However, NEP inhibitors may be implicated in the pathogenesis of AD through the increment of BK and NPs.

## 5 CONCLUSIONS

AD is a neurodegenerative disease characterized by intracellular accumulation of phosphorylated tau proteins and extracellular deposition of Aβ. Accumulation of Aβ may be due to defects in the proteolytic degradation and clearance, which occur mainly in late‐onset AD. Various protease enzymes, including NEP, are involved in the degradation and clearance of Aβ. Localization of NEP in presynaptic neurons facilitates clearance of Aβ. Thus down‐regulation of NEP is associated with Aβ accumulation, synaptic dysfunction, memory loss and progression of AD neuropathology.

On the other hand, NEP inhibitors which are used alone or in combination with ARBs in managing heart failure may increase AD risk by reducing Aβ clearance. According to preclinical studies, NEP inhibitors may increase AD development by augmenting Aβ accumulation. However, short‐ and long‐term clinical studies confirmed that LCZ696 had no serious adverse effects on cognitive function and AD risk in hypertensive patients. Furthermore, preclinical and clinical studies have revealed promising safety findings regarding the use of LCZ696. In addition, inhibition of NEP may lead to beneficial effects by regulating brain insulin sensitivity, inhibiting DPP4 expression and increasing GLP‐1 levels. Furthermore, BK which is also degraded by NEP is increased following the use of NEP inhibitors. In this state, increasing GLP‐1, BK and other substrate levels and inhibiting DPP4 expression may affect AD pathogenesis. Long‐acting GLP‐1 analogues are highly resistant to the effect of DPP4 which is mainly degraded by NEP, whereas DPP4 degrades endogenous GLP‐1. Endogenous GLP‐1 crosses BBB and plays a critical role in regulating cognition and neuronal growth. GLP‐1 inhibits brain oxidative stress and neuroinflammation; as well the cognitive enhancement effect of DPP4 inhibitors is related to the augmentation of GLP‐1. Thus, augmentation of brain GLP‐1 by NEP inhibitors may prevent AD development through modulation of brain glucose homeostasis, attenuation of brain IR, and mitigation of neuroinflammation and oxidative stress. Moreover, NEP inhibitors increase the level of substance P, which acts as a neurotransmitter and regulates diverse CNS functions, thus NEP inhibitors could effectively alleviate AD. However, NEP inhibitors may lead to detrimental effects on the cognitive and development of AD by increasing NPs and BK.

These findings proposed that NEP inhibitors may have a protective effect against AD development through increasing GLP‐1, NPY and substance P. However, NEP inhibitors may be implicated in the pathogenesis of AD through the increment of BK and NPs. The conflicting evidence concerning the role of NEP inhibitors in the progression of AD is not confirmed clinically. Therefore, additional preclinical and clinical studies are warranted in this regard.

## AUTHOR CONTRIBUTIONS

**Naif H. Ali:** Writing – review and editing (equal). **Hayder M. Al‐kuraishy:** Conceptualization (equal); project administration (equal). **Ali I. Al‐Gareeb:** Writing – review and editing (equal). **Saud A. Alnaaim:** Writing – review and editing (equal). **Athanasios Alexiou:** Supervision (equal); writing – original draft (equal). **Marios Papadakis:** Writing – original draft (equal). **Asmaa A. Khalifa:** Software (equal); visualization (equal). **Hebatallah M. Saad:** Writing – original draft (equal). **Gaber El‐Saber Batiha:** Resources (equal); writing – review and editing (equal).

## FUNDING INFORMATION

This work was supported by the University of Witten‐Herdecke Germany.

## CONFLICT OF INTEREST STATEMENT

The authors declare no conflict of interest.

## CONSENT FOR PUBLICATION

Not applicable.

## Data Availability

Data sharing is not applicable to this article as no new data were created or analysed in this study.
