# Correction to ‘Neprilysin inhibitors and risk of Alzheimer's disease: A future perspective’

**DOI:** 10.1111/jcmm.70058

**Published:** 2024-09-12

Ali NH, Al‐Kuraishy HM, Al‐Gareeb AI, et al. Neprilysin inhibitors and risk of Alzheimer's disease: a future perspective. *J Cell Mol Med*. 2024;28:e17993. doi:10.1111/jcmm.17993

In Introduction section, the sentence ‘AD is the most common type of dementia, accounting for about 70% of all dementia types’.^1^ is incorrect. The sentence should have read: ‘AD is the most common type of dementia, accounting for about 60% to 80% of all dementia types’.^1,149^

In Introduction section, the sentence ‘AD is the seventh leading cause of death in the United States, affecting 50 million people globally.^3^ Approximately 6% of the general population is affected, whereas more than 65% of affected cases are women; nonetheless, 10% of early‐onset dementia affecting people aged 30–60 is attributed to AD’.^3^ is incorrect. The sentence should have read: ‘The global incidence of all‐cause dementia is projected to rise from 50 million in 2010 to 113 million by 2050.^3^ Currently, over 55 million people suffer from AD, which ranks as the seventh leading cause of death in the United States.^150^ Additionally, women constitute over 65% of those affected by AD in the United States.^151^ Nonetheless, 10% of early‐onset dementia affecting people aged 30–60 is attributed to AD’.^3^

In the section 3 NEP INHIBITORS IN AD, the sentence ‘Sacubitril was the first NEP inhibitor approved in 2015 to manage heart failure’.^40^ is incorrect. The sentence should have read: ‘Sacubitril was the first NEP inhibitor approved in 2015 to manage heart failure’.^40,152^

Finally in Table 2 for the Bavishi et al.^43^ the study type ‘Experimental study’ is incorrect. The correct is ‘Review’.

**New References**

(149) (2023), 2023 Alzheimer's disease facts and figures. *Alzheimer's Dement*. 19: 1598‐1695. doi:10.1002/alz.13016

(150) 2024 Alzheimer's disease facts and figures. 1/6/2024. https://www.alz.org/media/Documents/alzheimers‐facts‐and‐figures.pdf

(151) Rabinovici GD. Late‐onset Alzheimer Disease. Continuum (Minneap Minn). 2019;25(1):14‐33. doi: 10.1212/CON.0000000000000700. PMID: 30707185; PMCID: PMC6548536.

(152) Fala L. Entresto (Sacubitril/Valsartan): first‐in‐class angiotensin receptor neprilysin inhibitor FDA approved for patients with heart failure. *Am Health Drug Benefits*. 2015;8(6):330‐334. PMID: 26557227; PMCID: PMC4636283.

We apologize for this error.

